# An easy method to differentiate retinal arteries from veins by spectral domain optical coherence tomography: retrospective, observational case series

**DOI:** 10.1186/1471-2415-14-66

**Published:** 2014-05-15

**Authors:** Yanling Ouyang, Qing Shao, Dirk Scharf, Antonia M Joussen, Florian M Heussen

**Affiliations:** 1 Charité-Universitätsmedizin Berlin, Department of Ophthalmology, Berlin, Germany

**Keywords:** Optical coherence tomography, Spectral domain, Retina vessel

## Abstract

**Background:**

Recently it was shown that retinal vessel diameters could be measured using spectral domain optical coherence tomography (OCT). It has also been suggested that retinal vessels manifest different features on spectral domain OCT (SD-OCT) depending on whether they are arteries or veins. Our study was aimed to present a reliable SD-OCT assisted method of differentiating retinal arteries from veins.

**Methods:**

Patients who underwent circular OCT scans centred at the optic disc using a Spectralis OCT (Heidelberg Engineering, Heidelberg, Germany) were retrospectively reviewed. Individual retinal vessels were identified on infrared reflectance (IR) images and given unique labels for subsequent grading. Vessel types (artery, vein or uncertain) assessed by IR and/or fluorescein angiography (FA) were referenced as ground truth. From OCT, presence/absence of the hyperreflective lower border reflectivity feature was assessed. Presence of this feature was considered indicative for retinal arteries and compared with the ground truth.

**Results:**

A total of 452 vessels from 26 eyes of 18 patients were labelled and 398 with documented vessel type (302 by IR and 96 by FA only) were included in the study. Using SD-OCT, 338 vessels were assigned a final grade, of which, 86.4% (292 vessels) were classified correctly. Forty three vessels (15 arteries and 28 veins) that IR failed to differentiate were correctly classified by SD-OCT. When using only IR based ground truth for vessel type the SD-OCT based classification approach reached a sensitivity of 0.8758/0.9297, and a specificity of 0.9297/0.8758 for arteries/veins, respectively.

**Conclusion:**

Our method was able to classify retinal arteries and veins with a commercially available SD-OCT alone, and achieved high classification performance. Paired with OCT based vessel measurements, our study has expanded the potential clinical implication of SD-OCT in evaluation of a variety of retinal and systemic vascular diseases.

## Background

Retinal blood vessels are the only visible and optically accessible small blood vessels in the human body [1]. In recent years, researchers have found that alterations in the arterial or venular tree of the retinal vasculature are associated with several generalized vascular problems, such as diabetic retinopathy, cardiovascular or cerebral disorders [2-7]. Since arteries and veins are differently affected in these disease processes, retinal imaging analyses like retinal vessel segmentation, quantitative vessel diameter measurement, or the ratio of artery to vein calibres (AVR) are of strong medical interest. A prerequisite for assessment of the above vascular change is to separate those vessels from each other [3].

Fluorescein angiography (FA) is a gold stand for classifying retinal arteries and veins, but due to its invasive nature, it is not employed merely for the purpose of differentiating arteries from veins. A number of methods have been reported in the literature for artery/vein classification of retinal blood vessels, which mainly depend on color fundus images [3,8-13]. Using color fundus image information, methods based on shape, colour and texture features, [11] local contrast of the red-channel [14] or red reflex [3,9-11,13-15], have great potential to classify the retinal arteries. However, such dissimilarities affect only the main vessels and strongly vary depending on patients, and vessel topography [11]. Also, the problem is complicated by the similarity in the descriptive features of these two structures and by the contrast and luminosity variability of the retina [8].

Optical coherence tomography (OCT) is an imaging modality capable of providing high-resolution cross-sectional images of the neurosensory retina. It has been described to provide superior morphologic information compared with colour photography and angiography [16-18]. The evaluation of retinal vessels by OCT has seen raised interest lately [19-28], of which Doppler Fourier-domain (FD) OCT was the most successful option [19]. However, Doppler FD-OCT systems are still several steps away from routine availability in clinic.

**Figure 1:**
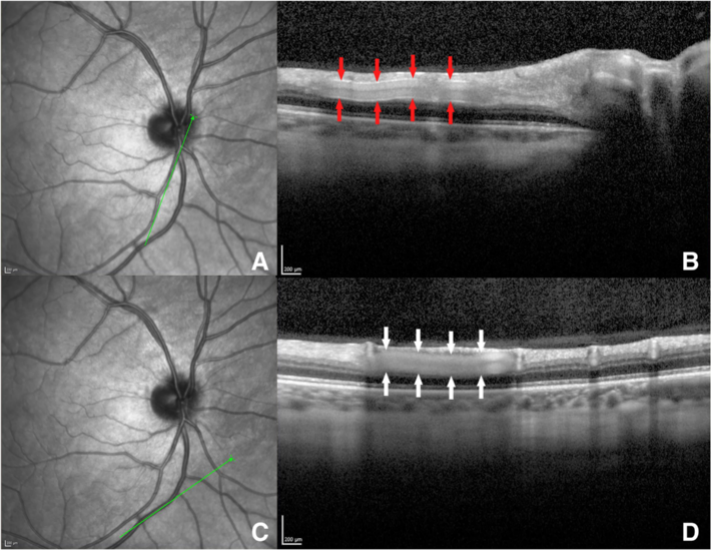
**One retinal artery and one vein seen on spectral domain optical coherence tomography. A**. Infrared reflectance image of a retinal artery. **B**. The same artery shown with optical coherence line scanning. Note: the upper and lower boundary of retinal vessels presented as hyperreflective feature compared to the surrounding retinal tissue (red arrows). **C**. Infrared reflectance image of a retinal vein. **D**. The same vein demonstrated with optical coherence scan. Note: the upper and lower boundary did not present different reflectivity compared to the surrounding retinal tissue (white arrows).

Recently, a spectral domain OCT (SD-OCT) based method for measuring retinal vessel diameters was reported using a commercially available spectral domain OCT (SD-OCT) instrument [22,29]. It has also been suggested that the hyperreflective core feature in retinal vessels seen by SD-OCT manifested differently between arteries and veins (as shown in Figure 1 in our preliminary data). Our study was aimed to evaluate an SD-OCT based method to differentiate retinal vessel types, and compare these findings with the reference from infrared images and/or FA.

## Methods

### Data collection

All Patients who underwent circular OCT-scans centered at the optic disc using a Spectralis OCT + HRA (Heidelberg Engineering, Heidelberg, Germany) at the Charité Hospital between January 1 2013 and March 1 2013 were retrospectively collected. Patients were seen in our primary care unit and did not necessarily present with retinal problems. Thus, eyes with relatively normal looking retinal structure in the OCT covered area were included. Clinical data regarding age, gender, race, history of ophthalmic diseases or surgeries, ophthalmic diagnosis, lens status, and visual acuity were also collected. Approval for data collection and analysis was obtained from the institutional review board of the Charité-Universitätsmedizin Berlin. Written consent was obtained from all patients. The research adhered to the tenets set forth in the Declaration of Helsinki.

The OCT scanning protocol consisted of a circular scan with 3.42 - 4.04 mm diameter centered on the optic disc (Spectralis HRA-OCT, Heidelberg Engineering, Heidelberg, Germany). The Eyes were scanned with the high-resolution OCT mode with a mean image averaging of 30 frames per final image. Simultaneously, near-infrared reflectance (IR) pictures were captured with a scanning laser ophthalmoscope. Images were reviewed and analyzed using the Spectralis viewing software (Heidelberg Eye Explorer, version 1.7.1.0).

### Grading methodology

One grader (Y.O.), certified for assessing OCT and color images at the Doheny Image Reading Center (DIRC), first reviewed IR images for every eye. Utilizing the Spectralis viewing software a circle representing the OCT scan was superimposed onto IR images. Vessels that did not cross or bifurcate at the intersection with the OCT scan were identified on IR and included in the study. Each vessel included by IR was given a unique identity number (ID) for subsequent grading [30]. Then the corresponding vessels on OCT were labelled with the same ID. An image of each vessel was made into a single frame (about 0.05 cm wide × 0.18 cm high, image resolution 300 pixel/cm) using Photoshop (version 6.0, Abode Systems, Inc, San Jose, CA, USA). This strategy allowed each vessel from different imaging modalities (IR or OCT) to be assessed in an independent, masked fashion without knowledge of its relationship with other vessels or eye.

After the initial vessel selection, two graders (Y.O., Q.S.) independently assessed the vessel type using IR and/or FA images by randomly selecting the vessel ID. However, the OCT images were covered during this step. Vessel types were labelled as “Artery”, “Vein” or “uncertain” by the maximum findings from IR and FA, and considered as ground truth for further analysis. In addition, vessel width obtained from IR (defined as minimum vessel diameter, measured vertically to the vessel axis with the caliper tool from the Spectralis software at the crossing point of vessel and OCT scanning line) was also evaluated for further analysis. The following brightness and contrast settings for IR images were used: “black on white” for color table, “medium” for sharpen and “none” for noise reduction.

**Figure 2:**
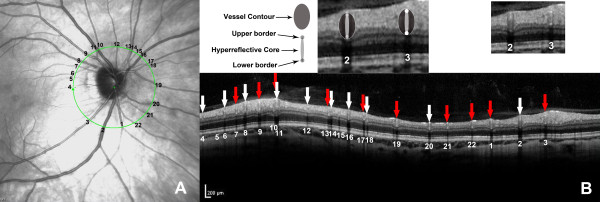
**Grading example of spectral domain optical coherence tomography based feature. A**. Infrared reflectance image with 22 vessels labeled. **B**. Optical coherence tomography circular scan centred at the optic disc. Retinal arteries (red arrows) and retinal veins (white arrows) were correctly classified based on the lower border reflectivity feature. Vessel No. 5 was classified incorrectly. Note: The image for each vessel is preferably assessed without magnification.

**Figure 3:**
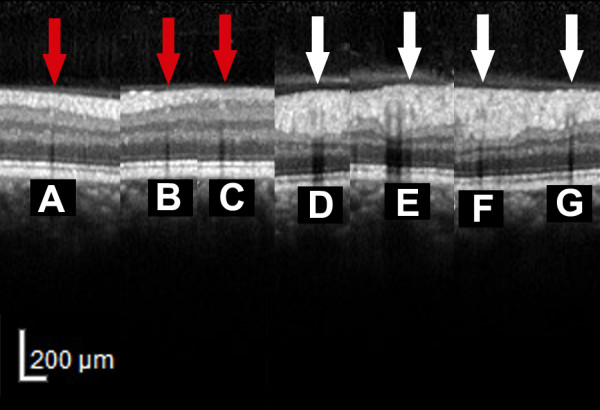
**Cases not applicable for grading of lower border reflectivity feature seen on spectral domain optical coherence tomography. A**-**C**: Red arrows: Small vessels without differentiable lower border reflectivity. **D**-**G**: White arrows: Vessels with lower border right at the boundary of hyperreflective and hyporeflective retinal layers.

The graders assessed each frame generated from the OCT images independently by randomly selecting the vessel ID. The structure of a vessel seen by SD-OCT is shown in Figure 2. For each vessel, a vessel contour was recognized in the inner retina. Within the contour, a hyperreflective core presented as equal or hyperreflective structure in comparison to the surrounding retinal tissue. The reflectivity feature at the lower border of the hyperreflective core (lower border reflectivity feature, LBRF) was used for SD-OCT assessment. The presence or absence of hyperreflective LBRF (HLBRF) was then documented as present (Y), absent (N) or cannot grade (NA) [31] (example shown in Figure 2). For this feature, vessels with a lower border right at the boundary of hyperreflective and hyporeflective retinal layers or small vessels without distinguishable lower border reflectivity were excluded due to the difficulty to interpret the true reflectivity features (example shown in Figure 3). In cases of disagreement, adjudication of the grading results by the senior grader F.H. took place to arrive at a common final answer [16]. The final agreement was calculated and used as final measurement for statistical analysis.

### Statistical analysis

Kappa values were used to assess the inter-grader agreement for vessel types obtained by IR and/or FA.

**Figure 4:**
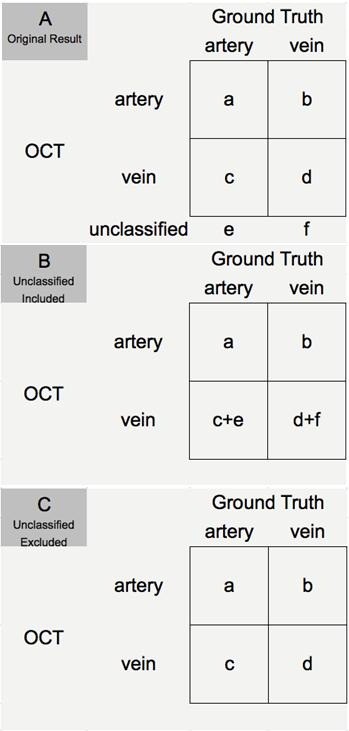
**Example of vessel type classification used for evaluation of classification performance determined by OCT and ground truth.** It demonstrates how we arrived at the numbers used to calculate sensitivity, specificity and other parameters mentioned in the methods from the original numbers **(A)** based on inclusion **(B)** or exclusion **(C)** of non-classified vessels.

OCT based features were considered corresponding to vessel types as follows: presence and absence of HLBRF was seen indicative for arteries and veins, respectively. When using vessel type as determined by the maximum finding from both FA and IR as ground truth, the criteria for evaluating classification performance for arteries were assessed in two ways: 1) unclassified vessels included: for all 308 vessels included in the study, considering the HLBRF as an indication of arteries, and absence of this feature (including “N” and “NA” for hyper reflectivity in the lower vessel contour) as negative result; 2) unclassified vessels excluded: only cases with gradable HLBRF were considered. Similar methods were used for veins. Then the classification performance was evaluated. For example from Figure 4, the classification performance of arteries (unclassified vessels included) was calculated as below:

$$\begin{array}{l} \mathrm{Sensitivity}=\mathrm{True}\;\mathrm{Positive}/\left(\mathrm{True}\;\mathrm{Positive}+\mathrm{False}\;\mathrm{Negative}\right)\\ \;=a/\left[a+\left(c+e\right)\right], \end{array}$$

$$\begin{array}{l} \mathrm{Specificity}=\mathrm{True}\;\mathrm{Negative}/\left(\mathrm{True}\;\mathrm{Negative}+\mathrm{False}\;\mathrm{Positive}\right)\\ \;=\left(d+f\right)/\left[\left(d+f\right)+b\right] \end{array}$$

$$\begin{array}{l} \mathrm{PPV}=\mathrm{True}\;\mathrm{Positive}/\left(\mathrm{True}\;\mathrm{Positive}+\mathrm{False}\;\mathrm{Positive}\right)\\ \;=a/\left(a+b\right), \end{array}$$

$$\begin{array}{l} \mathrm{NPV}=\mathrm{True}\;\mathrm{Negative}/\left(\mathrm{True}\;\mathrm{Negative}+\mathrm{False}\;\mathrm{Negative}\right)\\ \;=\left(d+f\right)/\left[\left(d+f\right)+\left(c+e\right)\right], \end{array}$$

$$\mathrm{Positive}\;\mathrm{Likelihood}\;\mathrm{Ratio}=\mathrm{Sensitivity}/\left(1-\mathrm{specificity}\right),$$

$$\mathrm{Negative}\;\mathrm{Likelihood}\;\mathrm{Ratio}=\left(1-\mathrm{Sensitivity}\right)/\mathrm{specificity},$$

$$\begin{array}{l} \mathrm{Classification}\;\mathrm{Accuracy}=\left(\mathrm{True}\;\mathrm{Positive}+\mathrm{True}\;\mathrm{Negative}\right)/\\ \;\left(\mathrm{True}\;\mathrm{Positive}+\mathrm{True}\;\mathrm{Negative}+\right)\\ \;\left(\mathrm{False}\;\mathrm{Positive}+\mathrm{False}\;\mathrm{Negative}\right)\\ \;=\left[a+\left(d+f\right)\right]/\left[a+\left(d+f\right)+b\right)\\ \;+\left(c+e\right)], \end{array}$$

Falsepositiverate=1−Specificity

$$\begin{array}{l} \mathrm{Classification}\;\mathrm{Error}\;\mathrm{rate}=\left(\mathrm{False}\;\mathrm{Positive}+\mathrm{False}\;\mathrm{Negative}\right)/\\ \;\left(\mathrm{True}\;\mathrm{Positive}+\mathrm{True}\;\mathrm{Negative}+\right)\\ \;\left(\mathrm{False}\;\mathrm{Positive}+\mathrm{False}\;\mathrm{Negative}\right)\\ \;=\left[b+\left(c+e\right)\right]/\left[a+\left(d+f\right)+b+\right)\\ \;\left(c+e\right)] \end{array}$$

As part of a subanalysis, similar analyses were also obtained by using vessel types documented by IR only as ground truth.

To compare differences of retinal vessel diameters between groups, univariate analysis of variance (ANOVA) was used. Correction for multiple testing was performed by post hoc Bonferroni adjustment.

Stata (version 10.0, College Station, TX: StataCorp LP. United States) was used for the statistical analysis. A bilateral value of P < 0.05 was considered statistically significant.

## Results

### Characteristics of the study population

Twenty-six eyes of 18 patients examined with the Spectralis OCT during the study period with the required scanning protocol were included in the study. Twenty-two eyes also had FA imaging covering field 1 and/or field 2 performed. Demographic features are summarized in Table 1.

**Table 1:** Demographic characteristics of the study

**No. patient (Women: Men)**	**18 (15:3)**
Age	58.7 ± 17.5 (23-85)
Mean ± SD (min-max), years	
Systemic Diagnosis Disease (No. Patients)	Diabetic (2), Hypertension (3), Other cardiovascular diseases (3), kidney stone (1), Thalassemia (1), Sarcoidosis (1), no systemic disease (8)
No. Eyes (Right: Left)	26 (13:13)
Snellen Visual Acuity min - max	20/200-20/20
Choroidal/Retinal Diagnosis Disease (No. Eyes)	Uveitis (8), Myopia (3), Diabetic retinopathy (1), Age-related macular degeneration (1), Juxtafoveal Telangiectasis (1), Macular pucker (1), Central serous retinopathy (2), Multifocal Choroiditis (1), Undetermined (2), within normal limits (6).

### Characteristics of vessel types by ground truth

A total of 452 vessels from 26 eyes of 18 patients with adequate image quality and without pathology within the OCT scan area were labelled in the study. Among them, 51 vessels were documented with unknown vessel type by using the maximum finding from IR and FA. Three more vessels were excluded due to bifurcation at the intersection of the OCT scan. Thus, a total of 398 vessels were included in the study, among which, 302 (75.9%) were classified by IR and additional 96 were only identifiable by FA.

### Classification of retinal vessel types by SD-OCT

**Figure 5:**
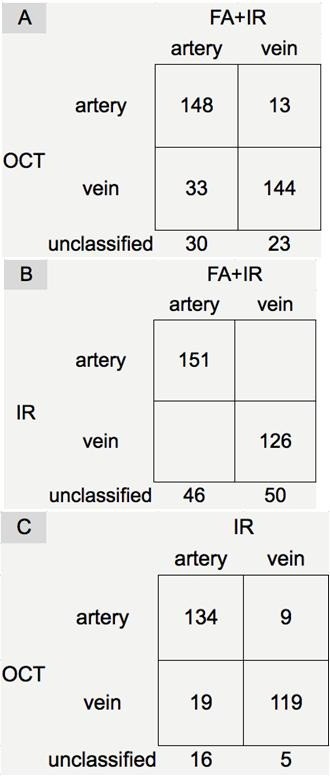
**Results of vessel type classification determined by OCT/IR and ground truth (FA + IR or IR).** This figure shows the numbers used to calculated specificity and sensitivity of OCT vs FA and IR **(A)**, IR vs FA and IR **(B)**, and OCT vs IR **(C)**.

Among the 398 included vessels, 27 vessels with their lower border right at the boundary of hyperreflective and hyporeflective retinal layers were documented as “NA” for the HLBRF. Additional 33 vessels also failed to have a differentiable reflectivity feature in the lower vessel contour due to the small size of the hypercore feature. As a result, 338 vessels were assigned a final grade for HLBRF in the study and differentiated into arteries and vein, of which, 86.4% (292 vessels) were classified correctly. 43 vessels (15 arteries and 28 veins) that IR failed to differentiate were correctly recognized by SD-OCT. 23 vessels crossed over one another another at the point of the OCT scan intersection and 21 (91.3%) of them were correctly classified. Classification performance parameters of the current method using FA + IR or IR only as ground truth are shown in Figure 5 and Table 2 (please note, the total number of vessels classified varied by using different ground truth).

**Table 2:** Classification performance parameters

**Performance measure**	**Using FA + IR as ground truth**				**Using IR as ground truth**			
	**Unclassified vessel**				**Unclassified vessel**			
	**Included**		**Excluded**		**included**		**Excluded**	
	**Artery**	**Vein**	**Artery**	**Vein**	**Artery**	**Vein**	**Artery**	**Vein**
Sensitivity	0.7014	0.8000	0.8177	0.9172	0.7929	0.8947	0.8758	0.9297
Specificity	0.9278	0.8436	0.9172	0.8177	0.9323	0.8876	0.9297	0.8758
Positive Predictive Value	0.9193	0.8136	0.9193	0.8136	0.9371	0.8623	0.9371	0.8623
Negative Predictive Value	0.7261	0.8318	0.8136	0.9193	0.7799	0.9146	0.8623	0.9371
Positive Likelihood Ratio	9.7120	5.1152	9.8751	5.0307	11.7173	7.9584	12.4561	7.4864
Negative Likelihood Ratio	0.3218	0.2371	0.1988	0.1013	0.2221	0.1186	0.1336	0.0803
Classification Accuracy	0.8056	0.8235	0.8639	0.8639	0.8543	0.8907	0.9004	0.9004
Classification Error Rate	0.1944	0.1765	0.1361	0.1361	0.1457	0.1093	0.0996	0.0996
False Positive Rate	0.0722	0.1564	0.0828	0.1823	0.0677	0.1124	0.0703	0.1242

Characteristics of vessel width for vessels that were correctly classified by IR + FA, IR and SD-OCT are summarized in Table 3. The mean vessel width was significantly different among groups with vessels determined by IR, vessels recognized by IR + FA, and vessels correctly differentiated by SD-OCT (ANOVA, p = 0.0008). The mean vessel width for vessels correctly classified by SD-OCT was not statistically different from the mean of those determined by IR (p = 0.348); or that recognized by IR + FA (p = 0.117). However, the mean vessel diameter for vessels determined by IR only was statistically smaller than those classified by IR + FA (P = 0.001).

**Table 3:** Characteristics of vessel width for vessels with correct classification

	**No. vessels with measureable vessel width**	**Vessel width (μm)**	
			**Mean SD**	**(min-max)**
All vessels by IR + FA	377	112.8 39.1	33-226
All vessels by IR	294	125.0 34.7	53-226
All Vessels by OCT	322	120.9 38.5	33-226
Arteries by IR + FA	190	105.0 28.0	35-180
Arteries by OCT	133	109.7 25.9	35-180
Veins by IR + FA	162	123.9 47.0	33-220
Veins by OCT	140	129.1 46.5	33-220

**Figure 6:**
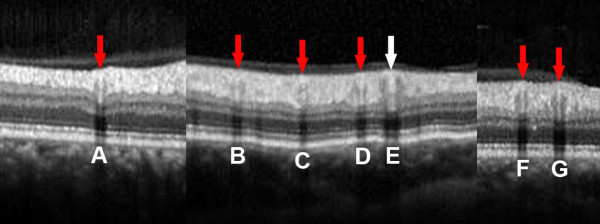
**False negative cases for spectral domain optical coherence tomography based vessel type. A**-**G**: Red arrows: Retinal vessel type determined by infrared reflectance and/or fluorescein angiography as arteries, but were incorrectly classified as veins by SD-OCT. White arrow: Retinal vessel type determined by infrared reflectance and/or fluorescein angiography as a vein, but were incorrectly classified as an artery by SD-OCT.

For cases that SD-OCT had classified incorrectly, 38 had measurable vessel width documented. Their mean vessel diameter was 100.2 ± 35.3 μm (range, 44–168). Case examples are shown in Figure 6.

### Inter-grader reproducibility

Almost perfect agreement [32] indicated by Kappa values was achieved for inter-grader reliabilities for vessel types by IR and/or FA (Kappa = 0.9306) and grading for the HLBRF by SD-OCT (Kappa = 0.8872).

## Discussion

Retinal arteries have clearly distinctive hyperreflective lower borders in SD-OCT but retinal veins do not. Using this feature, our study successfully differentiated retinal arteries from veins with a commercially available SD-OCT instrument.

OCT is commonly used in the diagnosis and management of retinal diseases, and is already a major non-invasive imaging modality in ophthalmology [19]. A recent report using commercially available SD-OCT has provided retinal vessel diameter measurements with good reproducibility [22]. The identification of retinal arteries and veins has to be done manually by comparing the OCT images with corresponding fundus images [22], which prevents OCT from being an independent useful tool to evaluate retinal vessels. However, retinal arteries and veins did exhibit different reflectivity patterns at close inspection (as shown in Figure 1 in our preliminary data). For arteries, both borders presented as hyperreflective in comparison to the surrounding retinal tissue; while for veins, no reflectivity difference was seen on either border compared to the surrounding retinal tissue. We chose the lower border of the hyperreflective core to be a potential indicator because the signal of the upper border is generally difficult to differentiate from the surrounding nerve fiber layer, ganglion cell layer, or inner plexiform layer. The lower border also has this limitation: when it is located near the border of hyperreflective and hyporeflective retinal layers, the assessment of its reflectivity is a challenge. However, this only occurred in 27 cases (6.8%) in our study.

Our study was the first to use a commercially available SD-OCT to classify retinal arteries and veins, and shows encouraging results. Previous literature for artery/vein classification of retinal blood vessels was based mainly on color fundus images [3,8,9,11,12,15,33,34]. Although other methodology, such as MRA was also used to separate arteries from veins, [35] it was more applicable for cerebral vessels. Our approach is not directly comparable to these publications. One reason is that virtually all of the previous reports used a vessel classification obtained by manual grading of colour images as ground truth (including only median or large vessels that can be recognized by colour images) and compared their (semi-) automatic classification system to this ground truth. To be possibly comparable to these reports, we utilized IR images and FA images as ground truth for vessel type. We also included rather small vessels, which potentially resulted in a lower classification rate compared to other reports. The other difficulty for comparison is that it is not clear in these reports if unclassified vessels were included in their sensitivity/specificity analyses. Thus, we performed analyses by both including and excluding the unclassified vessels in our study.

Our method achieved both high sensitivity and specificity for detection of retinal arteries: a sensitivity of 0.7929 (unclassified vessels included) or 0.8758 (unclassified vessels excluded) comparable with the methods from other groups, and a specificity of 0.93, higher than that reported by Saez et al. and Relan et al. [12,33]. The positive predictive value was 0.9371 for arteries. A false positive rate of 0.0677 or 0.0703 for arteries was also lower than in previous reports [12]. In addition, our system results in a higher positive likelihood ratio of 11.7173 or 12.4561 and equivalent negative likelihood ratio of 0.2221 or 0.1336 for arteries as compared to Saez et al. and Relan et al. [12,33] which confirms the high reliability of our proposed classification technique. Furthermore, the percentage of correct classification for arteries by our system was similar to those reported [10,33,36]. This good performance also held true for retinal veins. Altogether, our approach has shown high classification accuracy and specificity for both arteries and veins, which is at least analogous to the certified classification systems reported in the literature.

The current method has several potential advantages. Diametric to the classification method based on IR, which is vessel width dependent (larger vessels are most likely correctly classified), the current SD-OCT based technique could correctly classify both large and small vessels. The same point could be made from the descriptive data where the OCT based classification failed: vessels as small as 44 μm and as large as 168 μm were interpreted with a wrong vessel type. Thus, the current SD-OCT based method is more scanning angle or feature dependent. One additional advantage of the current method is that the classification of vessel types is not compromised at retinal vessel crossings. Generally, to correctly classify the retinal artery/vein at a crossing point is still a challenging task [9-11,13]. Our method was able to achieve 91.3% correct classification rate for the crossing vessels. Nevertheless, this new method has considerable limitations, of which we want to name the most important ones:

1. The system is sensitive to the quality of the images. If the image quality is not good enough, which is especially the case in the outer regions of the image - the reflectivity feature could not be assessed.

2. Vessels with a lower border right at the boundary of hyperreflective and hyporeflective retinal layers are limited by this method, because the true reflectivity features are difficult to interpret (example in Figure 3).

3. Some very small vessels without differentiable lower border reflectivity cannot be assessed (example in Figure 3).

The nature of the reflectivity difference between arteries and veins seen by SD-OCT is not known for certain. One explanation may be that the ultrastructural differences in the vascular wall of arteries and veins cause this difference in reflectivity. Interestingly though, one of the most important features for the discrimination of arteries and veins by colour fundus images is the central reflex in the red color channel [3,9,10]. It is caused by light reflection at the back of the vessel. The hypothesis is that because arteries carry blood rich in oxygen their inner part is brighter than their walls compared to veins, which makes the central reflex feature more obvious in arteries [3]. The same phenomenon may possibly explain our findings through altered reflectivity features. Still, this hypothesis needs to be verified in the future.

Our study has a number of limitations. Firstly, the differentiation of retinal vessels would strongly depend on image quality. However, this also holds true for the observation of other anatomical structures (especially for subtle changes), e.g. outer plexiform outer boundary, [37] or external limiting membrane. Secondly, due to the retrospective design, the reference vessel types were limited through the use of fundus images. Although FA was added in the assessment, it was done not specifically focusing on Field 1, so it only helped to differentiate 96 more vessels, leaving 51 with still unknown vessel type, which had to be excluded as ground truth. Using the same scanning protocol, prospective studies could be designed to include FA centered on the optic disc to enable better recognition of vessel types. In addition, further studies could aim to assess the performance of using SD-OCT based features to classify retinal vessel types based on different scanning patterns. Last and most importantly, some cases were classified incorrectly by the current method, as shown in Figure 6, the majority of which were arteries that were interpreted as veins due to the lack of the HLBRF. When the difference of the reflectivity feature is not obvious by manual grading, the approach failed. However, based on the current concept, a software-based grading system, which may better recognize the reflectivity difference, could potentially be designed by extracting the lower border reflectivity feature from targeted vessels.

## Conclusion

In summary, we were able to use an SD-OCT instrument to differentiate retinal vessel types with good classification performance. The findings from our study suggest that retinal vessel type assessment and diameter measurements can be achieved with the current generation of SD-OCTs and be routinely implemented in the clinic. Further studies could be designed to use the current easy and non-invasive method to evaluate a variety of retinal vascular diseases, or possibly systemic vascular disorders.

## Abbreviations

OCT: Optical coherence tomography; SD-OCT: Spectral domain OCT; IR: Infrared reflectance; FA: Fluorescein angiography; DIRC: Doheny image reading center; LBRF: Lower border reflectivity feature; HLBRF: Hyperreflective LBRF.

## Competing interests

Dr. Florian Heussen receives travel grants from the Allergan European Retina Panel. Dr. Antonia Joussen receives financial compensation for lectures from Novartis, Allergan Inc. and Bayer AG. The authors declare that they have no competing interests.

## Authors’ contributions

YO carried out the collection, analysis and interpretation of data; participated in drafting the manuscript. QS involved in data collection, analysis and revision of the draft. DS took part in data collection. AJ participated in its design and coordination and helped to revise the draft. FH was responsible for the preliminary thoughts and analyses, original study design, coordination and revision of the manuscript. All authors read and approved the final manuscript.

## Pre-publication history

The pre-publication history for this paper can be accessed here:

http://www.biomedcentral.com/1471-2415/14/66/prepub
